# Late-Onset COVID-19-Related Multi-System Inflammatory Syndrome in a Middle-Aged Man

**DOI:** 10.7759/cureus.15855

**Published:** 2021-06-23

**Authors:** Zubaida Al-Falahi, Salma Al-Harthi, Hatem Farhan, Ibrahim Al Busaidi, Abdullah M Al Alawi

**Affiliations:** 1 Medicine, Sultan Qaboos University Hospital, Muscat, OMN; 2 Internal Medicine, Oman Medical Specialty Board, Muscat, OMN; 3 Infectious Disease, Sultan Qaboos University, Muscat, OMN

**Keywords:** covid-19, multi-system inflammatory syndrome criteria, mis-a, sars-cov2, multi-system inflammatory syndrome in children (mis-c)

## Abstract

A 47-year-old man presented to the emergency department with persistent fever, chest pain and neck swelling, two months following a mild coronavirus disease 2019 (COVID-19) infection. He was found to have persistent fever, hypotension, cervical lymphadenitis, myocarditis, and acute kidney injury, collectively meeting the multi-system inflammatory syndrome criteria in adults (MIS-A). The patient responded well to methylprednisolone therapy and intravenous immunoglobulins with a complete clinical recovery. This case demonstrates that MIS-A can present as a delayed complication of COVID-19 infection.

## Introduction

In the aftermath of the novel severe acute respiratory syndrome coronavirus 2 (SARS-CoV-2) virus outbreak in December 2019, the world has been faced with significant social and economic disruption. SARS-CoV-2 is a single-stranded RNA virus belonging to the Beta group of coronaviruses. This virus targets angiotensin-converting enzyme 2 (ACE2) surface membrane proteins of nasal and bronchial epithelial cells and type 2 pneumocytes in the lungs through its viral spike protein [1]. The manifestations of the disease vary from mild symptoms to severe respiratory infection and systemic inflammatory response, causing multi-organ failure or death [2,3].

We present an unusual delayed extra-pulmonary manifestation that presented two months following a mild coronavirus disease 2019 (COVID-19) infection and known as a multi-system inflammatory syndrome in adults (MIS-A). In addition, we outlined the possible pathophysiology, clinical manifestations, and case management.

## Case presentation

A 47-year-old man presented to the emergency department with six days history of fever, chest pain, and neck swelling. The chest pain was central, radiated to the back and worsened on lying flat. Two months earlier, he had a mild COVID-19 infection that was managed symptomatically at home, and he did not receive COVID-19 vaccination. His medical background included diabetes mellitus, hypertension, and dyslipidemia, and his regular medications included took metformin, irbesartan, and simvastatin.

On presenting to the emergency department, the patient was alert and oriented. He was febrile (temperature 39 ˚C), tachycardiac (110 beats per minute), respiratory rate (RR) 20/minute, blood pressure (BP) 110/70 mmHg, oxygen saturation 97 on ambient air. Head and neck examination revealed bilateral cervical palpable lymph nodes. Cardiopulmonary examination revealed clear lungs with gallop rhythm on auscultation. Laboratory investigations are presented in Table 1. As summarised, there was neutrophilic leucocytosis with raised inflammatory markers, deranged coagulation profile, and acute kidney injury. In addition, he had raised troponin and significantly elevated brain natriuretic peptide (BNP).

**Table 1:** Laboratory test results during hospitalization and follow-up. WBC: white blood count; ANC: absolute neutrophil count; CRP: C-reactive protein; BNP: brain natriuretic peptide, eGFR: estimated glomerular filtration rate; INR: international normalized ratio; aPTT: activated partial thromboplastin time; PT: prothrombin time; HCO3: bicarbonate; CK: creatinine kinase; CK-MB: creatinine kinase-MB.

Test	Normal range	Day 1	Day 2	Day 4	Day 6	Day 16, OPD
WBC (10^9^/L)	2.2-10.0	18.7	17.9	-	18.3	14.6
ANC (10^9^/L)	1.0-5.0	16.4	16.6	-	14.3	11
Platelet count (10^9^/L)	150-450	134	113	165	307	263
CRP (mg/L)	0-5	365	-	97	47	-
Troponin (ng/L)	<14	602	252	168	150	18
BNP (pg/mL)	<124	22577	55297	23960	-	397
Creatinine (μmol/L)	59–104	214	219	150	124	92
eGFR (mL/min/1.73 m^2^)	>90	29	28	43	54	76
Sodium (mmol/L)	135-145	124	118	129	130	136
INR	0.91-1.09	1.35	1.21	-	1.07	-
aPTT (second)	26.4-38.1	91.1	69.5	-	27	-
PT (second)	12.8-17.6	14.4	13	-	11.6	-
D-Dimer (mcg/mL)	<0.4	-	2.6	-	-	-
Venous pH	7.35-7.45	7.363	7.278	-	-	-
Lactate (mmol/L)	0.5-1.6	2.6	2.9	-	-	-
HCO3 (mmol/L)	21.8-26.9	14	11	16	19	24
Anion gap	5.0-13.0	16	20	15	12	6
CK (U/L)	39-308	-	120	-	-	-
CK-MB (U/L)	<80	-	26.2	-	-	-
Ferritin (μg/L)	<30	-	654	-	-	-

Electrocardiogram (ECG) showed non-specific diffuse ST-segment elevation, and bedside echocardiography (ECHO) demonstrated global hypokinesia with severely depressed left ventricular ejection fraction of 30%, and there was no pericardial effusion. Ultrasound of the neck showed inflamed lymph nodes with findings suggestive of a suppurative lymphadenitis. The patient had normal liver function test.

The patient was admitted to high dependency unit, and he was started empirically on an adjusted renal dose of (piperacillin/tazobactam) for possible sepsis after collecting blood and urine cultures. However, a few hours later, the patient continued to deteriorate and developed worsening shock with tachycardia (126 bpm), hypotension (BP 90/60 mmHg), and tachypnea (RR 33 cycles/minutes). Given the rapid clinical deterioration with worsening cardiogenic shock and high clinical suspicion of a multi-system inflammatory syndrome triggered by COVID-19, the patient was treated with methylprednisolone pulse therapy (1000 mg) and intravenous immunoglobulins (IVIG) (0.5 mg/kg). In addition, diuretics and other heart failure medications were gradually started with close monitoring of the patient's hemodynamics.

**Figure 1:**
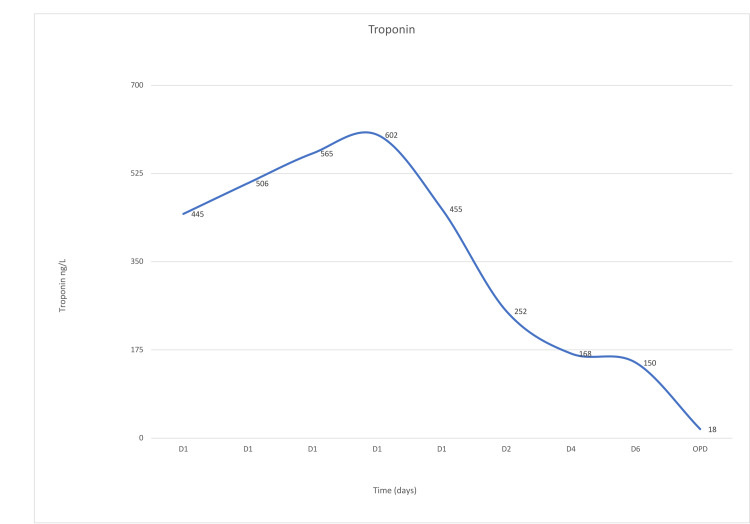
Trend of troponin values before and after initiation of the treatment.

**Figure 2:**
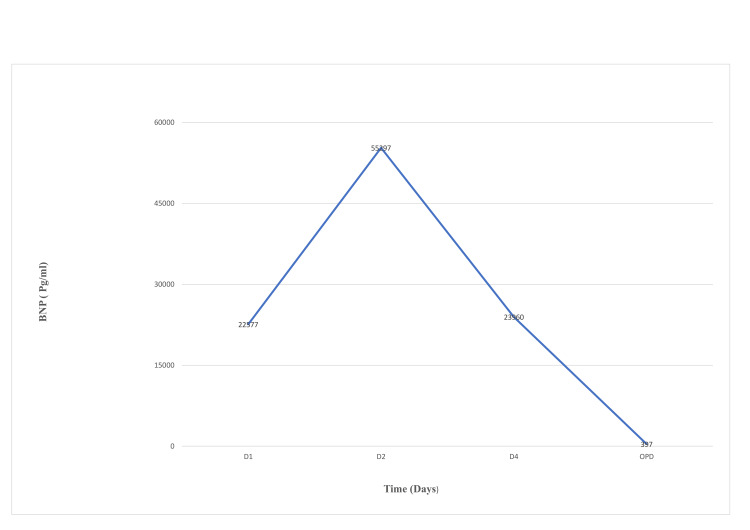
Brain natriuretic peptide (BNP) trends before and after initiation of treatment.

The patient responded well to steroid therapy, intravenous immune globulin (IVIG), and diuretics, evidenced by resolution of the chest pain, improvement of hemodynamics, the disappearance of heart failure signs (S3, bilateral basal fine crepitations), and declining levels of troponin (Figure 1) and BNP (Figure 2). Follow-up ECG showed resolution of ST-segment elevation. The renal functions and raised inflammatory markers reverted to baseline. Subsequent workup, including toxoplasma serology, urine legionella antigen, respiratory viral panel, blood and urine cultures, HIV serology, C3 & C4, and antineutrophil cytoplasmic antibodies (ANCA), were all negative.

After five days of hospitalization, the patient was discharged home on oral prednisolone (tapering schedule), lisinopril 10 mg once daily, bisoprolol 5 mg once daily, and frusemide 40 mg twice daily. The patient was reviewed in the medical outpatient clinic after two weeks, and he reported a remarkable symptomatic improvement. A follow-up ECHO showed normal left ventricular size and function (EF 61%). Moreover, follow-up laboratory tests showed remarkable improvement, as illustrated in (Table 1). 

## Discussion

Multi-system inflammatory syndrome in children (MIS-C) is a serious, rare, life-threatening condition characterized by a hyper-inflammatory state that affects multiple organs, including the heart, skin, gastrointestinal tract, kidneys, and other organs. MIS-C has been thought to resemble Kawasaki disease and toxic shock syndrome [4]. Diagnostic criteria of MIS-C include age less than 19 years, evidence of previous COVID-19 infection, fever for more than three days, involvement of at least two body organs, high inflammation markers, and the absence of active infection. All prior six criteria mentioned must be met for the diagnosis of MIS-C [5].

MIS is a new COVID-19 manifestation that is yet to be studied. Therefore, the exact mechanism by which the immune system is abnormally triggered remains uncertain; however, few theories could explain MIS. A possible cause of MIS-A is the dysregulation of host tissue's innate and adaptive immune system leading to multi-organ failure [5]. This is also thought to be due to the homology between SARS-CoV-2 spike protein and staph enterotoxin B super-antigen structure and sequence resulting in a hyper-inflammatory state [5]. SARS-CoV-2 binds to the angiotensin-converting enzyme 2 (ACE2) receptors in the vascular endothelium, which might explain endothelial damage and thrombo-inflammation complications. Another potential explanation is viral mimicry, which involves T-cells reacting to virally infected cells, forming immune complexes that trigger an inflammatory response or viral super-antigens that could trigger the immune system's cytokine-like storm [6].

It is thought that the host factors are the significant contributors to MIS rather than viral factors, according to a study done in London involving children infected with SARS-CoV-2 [7]. This can be translated into the adult population since they can present similarly. Among the limited, published cases on MIS, it was found that obesity was a common risk factor [8]. This could be attributed to the higher numbers of ACE2 receptors in adipose tissue, accumulation of inflammatory cells, and adipose tissue cytokines, which are pro-inflammatory. Afro-Americans/Caribbeans are somehow more prone to MIS for reasons that are not fully understood [9].

Severe COVID-19, by definition, is pneumonia along with evidence of hypoxemia. Critical COVID-19 is a feature of acute respiratory distress syndrome (ARDS) with or without the multi-inflammatory syndrome [10]. In contrast, MIS-A manifests with no or minimal pulmonary features, hypoxemia, or radiological evidence of pulmonary involvement [7]. In addition, MIS-A tends to have a temporal space from the initial COVID-19 infection, usually appearing at least two weeks after the initial infection. SARS-CoV-2 polymerase chain reaction (PCR) tends to be negative while serological tests are positive, indicating an element of seroconversion [11]. The time interval between covid infection and MIS-A development was two to five weeks in the previously published case reports. In our case report, the patient experienced MIS-A after 10 weeks of acquiring COVID-19 infection [11,12].

## Conclusions

COVID-19 infection can have delayed manifestations that affect extra-pulmonary organs. The resolution of acute COVID-19 symptoms, including mild infection, may not translate into resolved risk of complications. Awareness should be raised towards post-COVID-19 manifestations and complications for early detection to prevent the morbidities and mortalities associated with rare syndromes like MIS-A. MIS -C has been more recognized in children, but adults can present similarly, too.
